# Differential Consolidation and Pattern Reverberations within Episodic Cell Assemblies in the Mouse Hippocampus

**DOI:** 10.1371/journal.pone.0016507

**Published:** 2011-02-15

**Authors:** Remus Oşan, Guifen Chen, Ruiben Feng, Joe Z. Tsien

**Affiliations:** 1 Department of Pharmacology and Department of Mathematics and Statistics, Boston University, Boston, Massachusetts, United States of America; 2 Brain and Behavior Discovery Institute and Department of Neurology, MCG, Georgia Health Sciences University, Augusta, Georgia, United States of America; French National Centre for Scientific Research - University Paul Sabatier, France

## Abstract

One hallmark feature of consolidation of episodic memory is that only a fraction of original information, which is usually in a more abstract form, is selected for long-term memory storage. How does the brain perform these differential memory consolidations? To investigate the neural network mechanism that governs this selective consolidation process, we use a set of distinct fearful events to study if and how hippocampal CA1 cells engage in selective memory encoding and consolidation. We show that these distinct episodes activate a unique assembly of CA1 episodic cells, or neural cliques, whose response-selectivity ranges from general-to-specific features. A series of parametric analyses further reveal that post-learning CA1 episodic pattern replays or reverberations are mostly mediated by cells exhibiting event intensity-invariant responses, not by the intensity-sensitive cells. More importantly, reactivation cross-correlations displayed by intensity-invariant cells encoding general episodic features during immediate post-learning period tend to be stronger than those displayed by invariant cells encoding specific features. These differential reactivations within the CA1 episodic cell populations can thus provide the hippocampus with a selection mechanism to consolidate preferentially more generalized knowledge for long-term memory storage.

## Introduction

The hippocampus plays a crucial role in converting recent episodic events into long-lasting memories, a process termed memory consolidation [1]–[5]. While our brains can recall a great amount of detail immediately after the event (within the time domain of short-term memory), there appears to be a gradual loss of many specific details in the domain of long-term memory [1], [6], [7]. In other words, long-term memory eventually contains only partial information about the original experiences, usually retaining more general and abstract information rather than a full set of specific details. Two major possibilities could underlie such biased memory storage processes: 1) the brain somehow preferentially consolidates general information over specific information (selective consolidation hypothesis); 2) both general and specific information are initially equally consolidated, but specific details somehow degrades more easily over time than does general information (degradation hypothesis). In the present study, we investigate if and how the hippocampus may engage in differential consolidation of memory patterns that were triggered by robust episodic events.

Since the hippocampus is well known for its crucial role in converting an episodic memory from short-term into its long-term form, it is of great interest to use episodic memory paradigms for the identification of memory traces in its networks [8], [9]. Investigating the neural mechanism of episodic memory consolidation can be approached by examining the activity replay in the hippocampus. For example, large-scale recording and decoding methods show that the real-time encoding patterns seem to reappear in the hippocampus within seconds-to-minutes after the animals encounter startling or emotionally charged episodic events [10], [11] or fear conditioning [12]. Moreover, it has been shown in the trace fear conditioning paradigm that conditioned tone responses and tone-shock association patterns undergo trial-dependent increase in the numbers of replay during learning, correlating tightly with increased immediate freezing [12]. This is the first evidence that links memory pattern replay with behavioral performance scores [12]. In addition, it seems that a significant fraction of pattern replays are associated with ripples [12] which may be related to memory consolidation [13]–[15]. Studies in using place cells with overlapping place fields also suggest reactivations after running [16]–[20], although the relationship between such place cell replays and spatial memory is unclear. Nonetheless, the various observed pattern replays is, in general, consistent with the explanation of its potential roles in memory consolidation.

To our knowledge, however, there is no report aimed at addressing the following important question: how and why does the hippocampus only convert a fraction of original information into long-term memory? In the present study, we set out to investigate how the distinct cell populations in the CA1 region of the hippocampus may engage themselves during the post-learning consolidation of episodic experiences. We used a set of fearful episodic events, coupled with large-scale neural ensemble recording methods [10], [11], [21], for investigating episodic memory consolidation mechanisms. We report that the CA1 cells which encode different aspects of episodic events, tend to be reactivated differently during the post-learning period.

## Results

### Organization of CA1 cell assemblies in responses to different robust episodes

**Figure 1:**
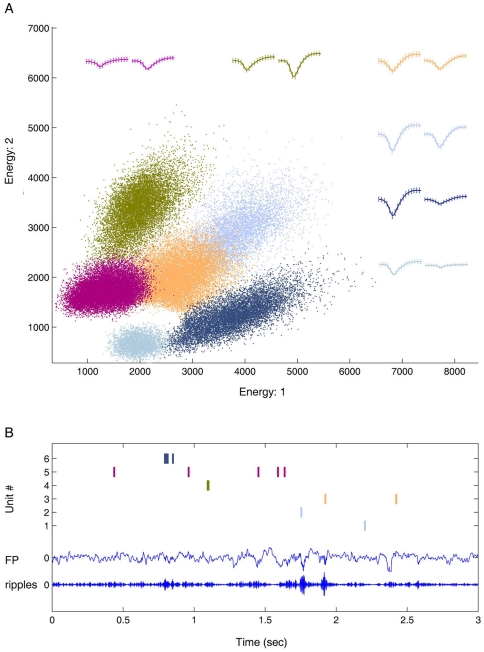
Separation of multiple single units by a single stereotrodes. (**A**) Six single units were detected by a single stereotrode. (**B**) The spike raster plot for the corresponding six units was shown together with the original field potential and the filtered ripple signal from one channel.

**Figure 2:**
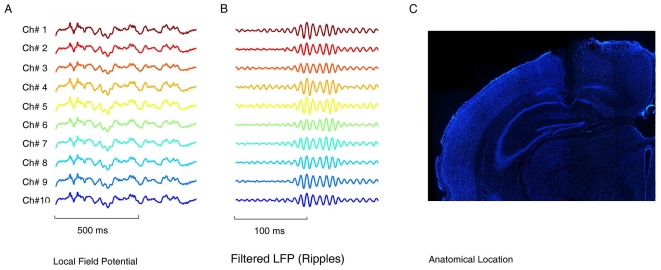
Evidence for confirming the position of recording electrodes in CA1. (**A**) Local field potentials from ten separate recording electrodes. (**B**) Filtered ripples from the corresponding ten electrodes. (**C**) Histological confirmation of electrode placement. Nissl-staining coronal section through the CA1 field of the hippocampus shows the position of the electrodes.

**Figure 3:**
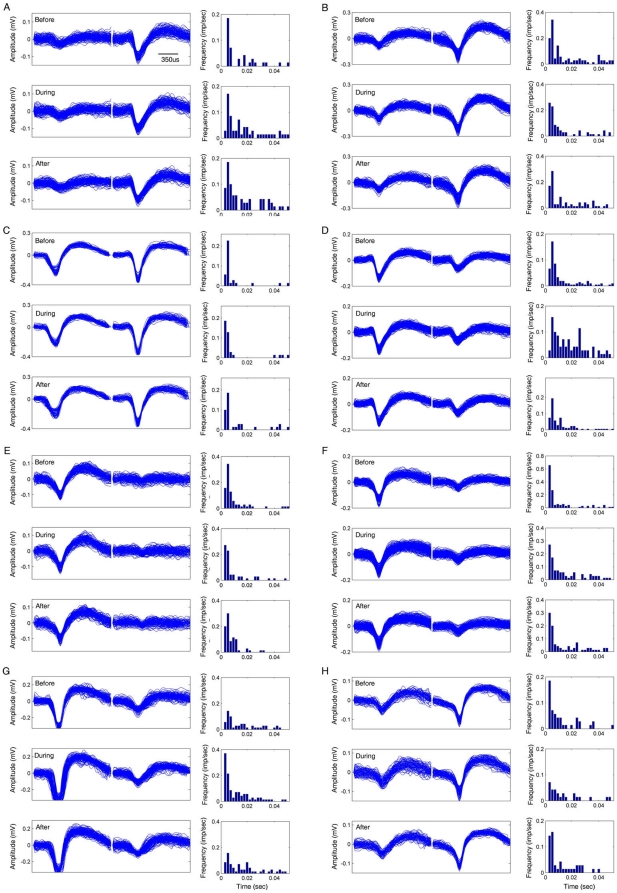
Stable recordings of pyramidal cells, as confirmed by the waveforms and inter-spike interval (ISI) histograms. Eight representative putative pyramidal cells are shown here. The left columns are waveforms and the right columns are inter-spike interval histograms. The waveforms were plotted during 70-sec recordings before (top row), during (middle row), and after trace-conditioning trials. A 10-sec recording for each trial was plotted. The ISIs were analyzed by using the corresponding data and the bin size is 0.005 s.

**Figure 4:**
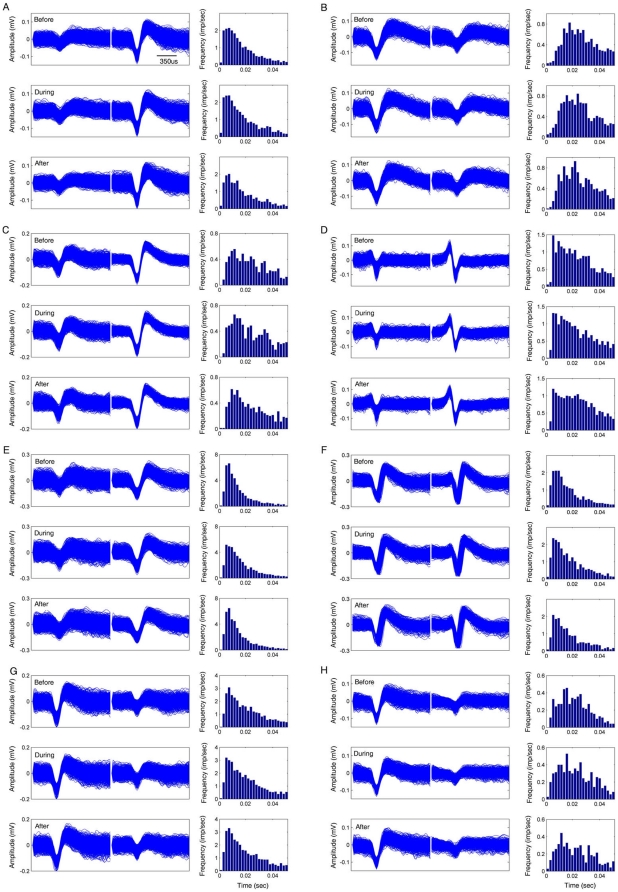
Stable recordings for putative interneurons in the hippocampus. Waveforms and inter-spike interval histogram of interneurons (eight representative units) are presented here. The left columns are waveforms and the right columns are inter-spike interval histograms. The waveforms were plotted from a 70-sec recording before (top row), during (middle), and after trace-conditionings (bottom row). A 10-sec recording for each trial was plotted. The ISIs were analyzed using the corresponding data and the bin size is 0.005 s.

**Figure 5:**
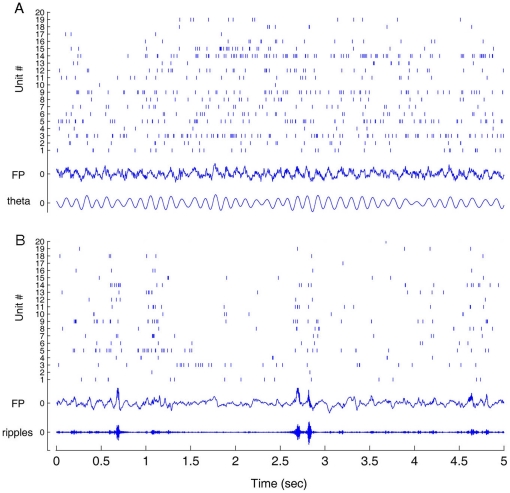
Simultaneous recordings of many individual units (only 20 units shown here out of over a few hundreds) and local field potentials in a freely behaving mouse were used for assessing whether our electrodes reached CA1. (**A**) The activity of the simultaneously recorded individual units from the hippocampus during mouse exploration. Twenty units from the recorded data were selected for the illustration. Note that the simultaneously recorded field potential shows the typical theta rhythm oscillations (4–12 Hz) during running. (**B**) The activity of the simultaneously recorded individual units from the hippocampus during mouse slow wave sleep. The simultaneous field potential recording shows the irregular waves as well as the ripple oscillations (150–250 Hz) during sleep. The traces marked with ‘FP’ show the original field potential recorded from one recording channel. The trace marked with ‘theta’ shows the field potential filtered with the frequency range from 4–12 Hz from the original one, whereas the traces marked with ‘ripples’ shows the field potential filtered with 150 to 250 Hz from the original one.

To investigate CA1 neural activity patterns during and after learning period, we exposed naive mice to four types of the fearful episodic events: 1) A sudden drop of animal in a small elevator (Elevator-Drop); 2) A sudden air-blow to animal's back (Air-Blow); 3) A sudden earthquake imitated by shaking the animal in its cage via vortex machine (Quake); and 4) A startling acoustic sound (Sound). Using a 96- or 128-channel Plexon neural data acquisition system, we recorded bilaterally from the dorsal region of hippocampus using a microdrive with adjustable stereotrodes as described previously [10], [12], [21]. Recorded units were spike-sorted (Figure 1). Only units with clear boundaries and less than 0.5% of spike intervals within a 1 ms refractory period were included in the present analysis. The location of electrode bundle tips in CA1 was confirmed by physiological markers through the occurrence of sharp-wave associated ripples (100–200 Hz) as well as by histological staining of the post-experiment brain sections (Figure 2). Based on spike waveforms, firing rates, and inter-spike intervals, the recorded CA1 units were divided into two classes: principal excitatory units (putative pyramidal neurons) (Figure 3) and inhibitory units (putative interneurons) (Figure 4). Putative pyramidal cells are characterized by lower firing rates, wider waveforms, and complex bursts with 2–10 ms inter-spike intervals, reflected by their autocorrelograms (Figure 3). In addition, we calculated the complex spike index, defined as the percentage of spikes with first lag inter-spike intervals that fall between 2 and 15 msec and whose second spike is smaller than the first. The averaged complex spike index from these recorded pyramidal cells was 14.9±0.63. On the other hand, putative interneurons are characterized by higher discharge rates, narrower waveforms, and autocorrelograms with a much slower decay (Figure 4). Simultaneous recordings of local field potentials also exhibited characteristic theta oscillations during running (Figure 5A) or high frequency ripples during slow wave sleep (Figure 5B), confirming the CA1 location of our electrodes. In general, pyramidal cells constitute the majority of the recorded cells in the CA1 region. The stability of the recordings was also confirmed by the near identical waveforms of the units before, during, and after the various startling events (see the top, middle, and bottom insets of each subpanel in Figure 3 and 4).

**Figure 6:**
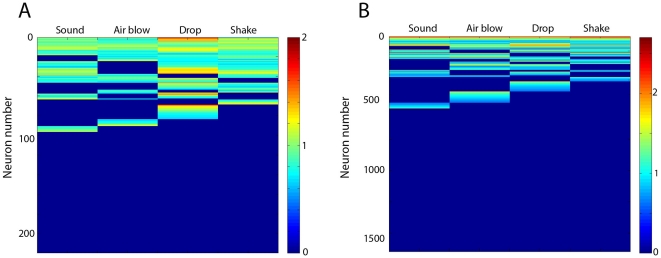
Hierarchical organization of CA1 cell assemblies from general-to-specific response-selectivity. (**A**) Hierarchical clustering for simultaneously recorded 219 neurons (mouse #1) suggest a wide range of response selectivity to startling stimuli, ranging from general (top of the figure, responsive to all four type of startles), to sub-general (responses to a subset of two or three types of events), highly specific (1 type) and non-responsive units (bottom of the figure, in blue). The following formulas have been used during the hierarchical clustering procedures: the average responses to a startle R_startle_ were first normalized to R_normalized_  = (R_startle_ – R_basal activity_)/(R_average_ + R_basal activity_), where the average population activity during baseline activity is R_average_ ≈2.7 To facilitate visualization of different classes, we retain only the units that have a positive change in their firing rates, and we display the quantities T =  log(1+ R_normalized_) as a colormap. (**B**) Although exact percentages for these types of units may vary from animal to animal depending on the location of electrodes, pooled data from 7 animals (1623 neurons) indicate that this is a general property.

To efficiently deal with the large datasets, we employed hierarchical clustering analysis to examine the firing responsiveness of those units among all of the recorded mice. Our analysis shows that each specific event is represented by a set of CA1 cell assemblies, or neural cliques, that respond with a range of selectivity, from the general response to all four events to the specific response to a single event (Figure. 6A, data from one mouse), although, we note here that significant subpopulations were unresponsive to any of those fearful events. As a result, the hippocampal responsive cells can be classified based on their response selectivity for different types of episodic events. For example, we found that a large number of cells exhibited broad responses to all types of episodic stimuli including the elevator-drop, earthquake, air-blow, and loud sound. These cells were termed as general responsive cells, or the general clique (top rows in Figure 6A). In the case of the sub-general cells or subgeneral cliques, they responded to a combination of two or three types, but not to all of the episodic events. In addition, there were groups which exhibited high specificity towards one specific type of event (middle rows with one response per category in Figure 6A). In agreement with the simultaneously recorded data [10], the pooled dataset from all of the recorded mice again show the existence of this overall hierarchical arrangement in CA1 cell response selectivity (from general to specific-response features) (Figure 6B).

### Encoding of episodic events by intensity-sensitive cells and intensity-invariant cells

While many CA1 cells changed their firing rates in response to external inputs or experiences, it is not clear to what degree the activations of hippocampal cells by such episodes reflect memory encoding or merely represent sensory inputs. Our hypothesis is that the neural responsiveness should reflect changes in input intensities (intensity-sensitive neurons) if it is mere representation of sensory inputs. On the other hand, some neurons in CA1 may exhibit fairly equal firing responses despite changes in event intensities (Intensity-invariant neurons) because they may be more geared towards the encoding of invariant important features.

**Figure 7:**
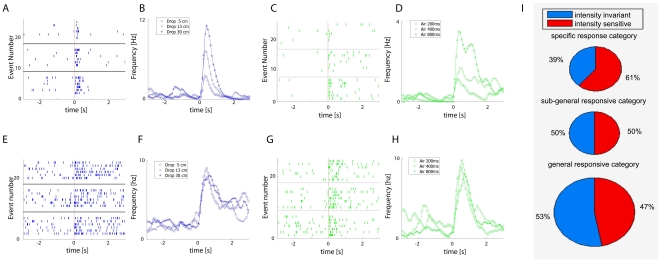
Effects of event-intensity on CA1 cell responsiveness. (**A**) A representative CA1 unit shows intensity-dependent modulation of its firing changes in response to different drop heights from 5, 13 and 30 cm (upper, middle and lower raster, respectively). Time is represented on the horizontal X-axis (−3 to 3 seconds) and the trial number is listed on the vertical Y-axis. The vertical red line indicates t = 0. (**B**) The frequency responses of the same unit (shown in A) obtained by smoothing the spike count through an asymmetric kernel indicate that this neuron increases its firing rate monotonically in response to drop heights. (**C**) Spike rasters of a CA1 cell that show a intensity-dependent unit which increases its firing in response to various amount of air-blow (for the durations of 200 ms, 400 ms, and 800 ms (upper, middle and lower panel, respectively). (**D**) The frequency responses obtained by smoothing the spike count through an asymmetric kernel indicate that this CA1 neuron increases its firing rate monotonically over change of stimuli amounts. (**E**) A representative CA1 unit that exhibit invariant firing increase over changes of drop heights (from 5, 13 and 30 cm; upper, middle and lower raster, respectively). (**F**) Smoothed frequency responses confirm that this neuron is invariant to drop heights. (**G**) Spike rasters for a representative neuron that responds in an invariant fashion to air-blow stimuli. (**H**) Smoothed frequency responses indicate that this CA1 neuron responds in an invariant fashion to the amount of air being blown. (**I**) The percentages of units that belong to specific, subgeneral and general populations are represented schematically by the size of the corresponding circles. 0.20 = 20%. 0.26 = 26%, and 0.54 = 54%. In addition, the partition between intensity-invariant and intensity-sensitive are displayed in light blue and orange-red.

Thus, we conducted a series of parametric experiments and varied the level of intensity of the two episodic events (drop and air blow). We set the dropping height of at 5 cm, 13 cm and 30 cm, respectively, for the drop; adjusted airflow at 200 ms, 400 ms and 800 ms, respectively, for the air blow. Our parametric experiments reveal the existence of two major types of responsive cell groups in CA1, namely, intensity-sensitive responsive neurons and intensity-invariant responsive neurons. The input-sensitive group contains units that either increase or decrease their firing rates in a monotonic fashion with changes in the amount of stimulus inputs (Fig. 7A and 7B for an example of a drop intensity-responsive neuron, and Fig. 7C and 7D for an example of an air-blow intensity-responsive neuron). In contrast, intensity-invariant responsive neurons are characterized by similar changes in their firing rates irrespective of the magnitude of the stimulus inputs (see Fig. 7E and 7F, for an example of a drop intensity-invariant neuron; and Fig. 7G and 7H for an example of an air-blow event-invariant neuron, respectively). It is noteworthy to point out that these episodic events can trigger firing changes in the vast majority of both intensity-sensitive and intensity-invariant cells independent of the animal's specific location within a given environment and the locomotor states of animals (such as running or in rest). For example, during the course of repetitions of air-blow or shake, the mice usually moved from one location to another and change their locomotion behavior, say from quiet wakefulness, to running, glooming, or exploring, etc. This suggests that the effectiveness of startling episodes in triggering CA1 responses is not constrained by specific place location or locomotory state of the animals [10], [12], [22]. There is, however, a small number of cells whose firing changes are dependent on both the event and the overall environment in which the event took place (thereby, reflecting the integration of both event and contextual information) [10].

From a total of 1623 units recorded from 7 mice, 583 units (35.9%) responded to various startling stimuli. Of them, 284 units belong to the intensity-invariant cell group and 299 units exhibit intensity-sensitive changes of their firing rates, close to 1∶1 distribution ratio. We have further analyzed the interaction between the intensity-sensitivity categories vs. event-response selectivity categories. We found that percentages of invariant neurons that belong to general to specific categories are: 54% for the general-responsive category (152 out of a total of 284 intensity-invariant neurons), 26% for the sub-general responsive category (75/284), and 20% for the specific event-encoding cells (57/284). Similarly, the percentages of intensity-sensitive neurons that belong to general, subgeneral and specific modulated neurons are 45% (134/299), 25% (75/299) and 30% (89/299), respectively (Figure 7I).

### Stronger event-intensity produces better pattern separation

**Figure 8:**
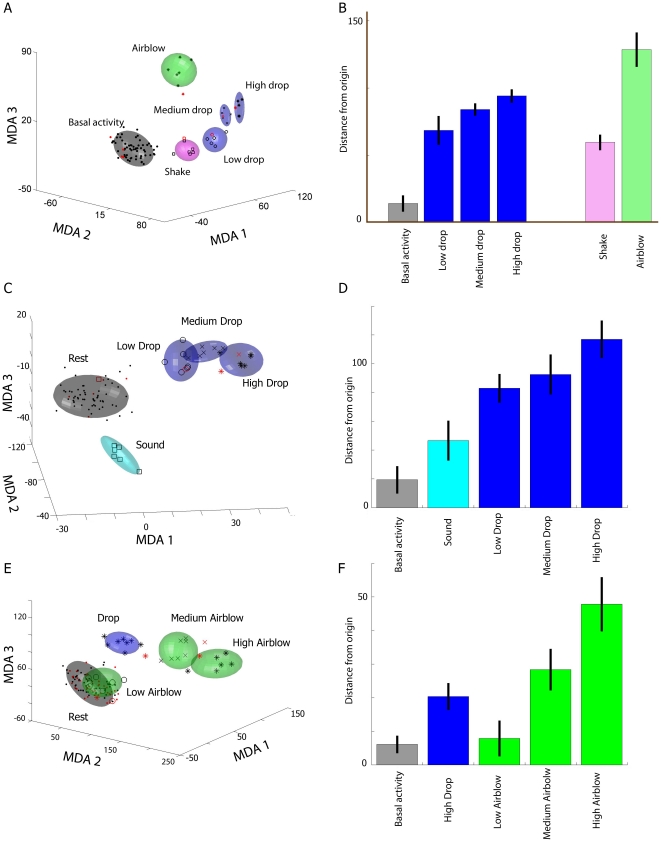
CA1 ensemble pattern classification. (**A**) MDA analysis on mouse #1 shows that CA1 ensemble representation of high drop experiences is located farther away from the resting state in the projection subspace than the clusters corresponding to low or medium drop episodic startles. (**B**) Quantification of the average distances away from the rest origin for all episodic events plotted in panel **A** illustrates that classification of higher drops are indeed located further away from the resting state in the encoding subspace. (**C**) A data set from another mouse hippocampus (mouse #2) is characterized by a large separation between high drop (third type of drop from a 30 cm height) and the basal activity cluster, when compared to the separation between low drop (first type of drop from a 5 cm height) or medium drop (second type of drop from an 11 cm height). (**D**) Inspection of the average distances away from the origin of the basal activity (gray), air-blow (light blue) and drop clusters (dark blue) confirm with the trends suggested by panel C. (**E**) Results from a third data set (mouse #3) indicate that while air-blow at low intensity evoked little ensemble response, air-blow at middle and high intensity evoked considerably larger responses. (**F**) Quantification of distances away from origin by various clusters is in agreement with panel E. Overall, these three examples illustrate the general tendency from all our data sets: the stronger the parametric stimulus, the better the separation from rest cluster in encoding subspace.

To seek a statistical description of ensemble neural activity patterns from the recorded large datasets, we employed Multiple Discriminant Analysis (MDA) which has been shown to be an effective method for statistical pattern classifications of large neural data collected from well defined event categories [10], [12], [22], [23]. Using this method, we find that these ensemble activity patterns corresponding to these four different episodes can be quantitatively classified and intuitively visualized as distinct ellipsoid clusters in MDA subspaces (Figure 8). By and large, the clusters corresponding to lower-intensity episodic stimuli are situated closer to the basal activity class, while the highest-intensity clusters are located furthest away. The intermediate classes often lie in between (see Figure 8 for results from three mice that include parametric changes in drop and air-blow intensities, Data from Mouse#1 is presented in A and B; Mouse#2 in C and D, and Mouse#3 in E and F, respectively). Our analysis of all data further shows that this is a general trend across all seven recorded mice (See Table 1, where absolute distances were normalized by the standard deviation corresponding to the Rest cluster to allow for uniform comparison across multiple data sets).

**Table 1:** Distance from the rest origin for various event clusters.

Data set	Acoustic sound	Air 200 ms	Air 400 ms	Air 800 ms	Shake	Drop 5 cm	Drop 11 cm	Drop 31 cm
Mouse #1	2.41	3.09	5.24	6.64	3.78	3.87	7.55	8.02
Mouse #2	1.58	0.86	2.14	2.42	2.29	1.52	2.16	3.61
Mouse #3	0.91	0.25	2.88	4.11	5.48	1.54	2.07	5.82
Mouse #4	3.35	0.75	1.78	1.54	5.11	5.83	6.20	6.82
Mouse #5	1.51	1.33	3.35	4.13	2.71	1.23	1.56	2.44
Mouse #6	0.84	0.82	2.56	3.08	1.55	2.88	2.98	5.17
Mouse #7	0.61	1.02	2.81	3.70	3.03	4.55	3.56	4.66

### Transient dynamics of CA1 ensemble traces

**Figure 9:**
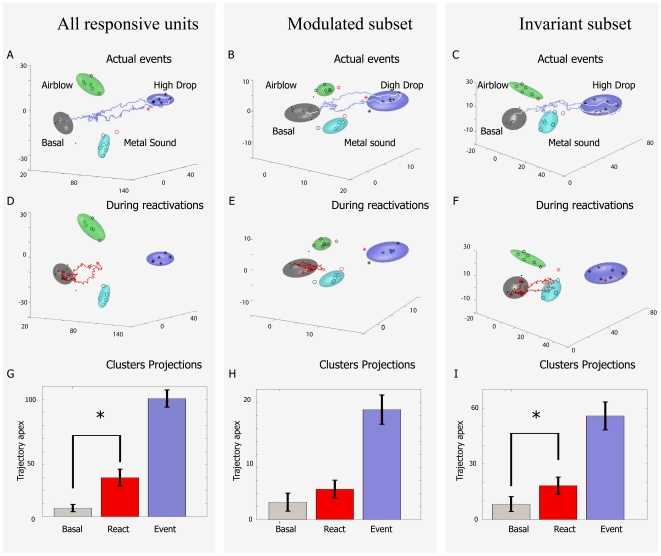
Pattern reactivations are mainly driven by the invariant subpopulation. (**A**) A typical trajectory during a drop event from 30 cm (gray/cyan/green/blue clusters indicate rest/sound/air 800 ms/drop 30 cm) is plotted using all of the responsive units. (**B**) Activation dynamics can be also observed in the MDA encoding subspace which uses only the modulated subpopulation of cells. (**C**) Activation dynamics can be further observed in the MDA encoding subspaces constructed from the invariant subpopulation only. (**D**) A typical reactivation trajectory is detected in whole population activity. (**E**) However, at this time point little reactivation is observed in the intensity-modulated subpopulation. (**F**) In contrast, the invariant responsive subpopulation exhibits a significant reactivation. Please note that the directionality of trajectory towards the drop cluster and away from the air-blow cluster or acoustic metal sound is confirmed in other rotated 3-D dimensions. (**G**) Comparison of distances from the resting state for all reactivation occurrences when using all of the responsive units reveals significant differences between basal states and reactivations. (**H**) No statistically significant reactivations in the intensity-modulated subpopulation. (**I**) Significant reactivations are observed in the intensity-invariant subpopulation (p<0.05).

To dynamically monitor the population firing patterns, we applied a sliding-window technique to the MDA method which enabled us to directly visualize the real-time network-level memory encoding dynamics [10], [12], [22]. Using the fixed matrix coefficients produced by the MDA method, we computed the instantaneous projection of neural responses during the entire experiments (using two 500-msec width-bins, sliding at 10-msec time resolution). As such, the temporal evolution of the ensemble activity patterns can be directly visualized as dynamical trajectories in the encoding subspace [22]. For example, during the baseline state prior to a drop event, the instantaneous projection was confined to the Rest ellipsoid. Upon the sudden drop, however, we observed a planar trajectory that began in the Rest cluster, quickly visited the corresponding Drop cluster and then returned to Rest (an example for Drop is shown Figure 9A). By separately calculating the dynamical evolution of the neural activity in the encoding subspace, we found that both types of CA1 responsive cells (namely, input intensity-sensitive cells and input intensity-invariant cells) can produce robust event-encoding trajectories of the startling episode (Fig. 9B and 9C - note that these two encoding subspaces are computed based on the input obtained after partitioning data into two subsets). This indicates that intensity-sensitive and intensity-invariant cells all contribute to the CA1 ensemble classification and representation of the actual event at the time when it happens.

By scanning through the recorded CA1 neural activities in the post-event period, we observed that these transient encoding patterns triggered by startling stimuli reactivated spontaneously as indicated by dynamical trajectories with similar geometric shapes but at smaller amplitudes (Figure 9D). These reappearances of transient trajectories usually occurred within several seconds to minutes after the actual events, in agreement with our previously published research [10]–[12]. We note here that each one of the episodic events may be followed by these spontaneously-emerging patterns and that no discernible pattern can be observed regarding the timing of these putative reactivations that presumably are related to the processing of the newly-acquired memory traces. More precisely, these reactivations do not require the presentation of a full sequence of events in order to become manifest, instead they may arise immediately following the first episodic event. By taking advantage of the ability of using the MDA encoding subspace to monitor the neural ensembles dynamics during whole duration of the experiments, we applied a sliding window technique to compute the projection of neural activities to identify the exact time point at which reactivations took place. As a result, our identification of the putative memory reactivation is determined by MDA analysis. We then asked how intensity-invariant and intensity-modulated neurons would contribute to the ensemble pattern classifications and representations during events and during post-event reactivations. Interestingly, our analysis revealed that the intensity-sensitive (intensity-modulated) cell population, by and large, exhibited only negligible reactivation or no reactivation at all (Fig. 9E). On the other hand, the invariant cells seemed to produce reliable and substantially larger reactivation trajectories (Figure 9F). This sub-categorical analysis thus indicates that the invariant cell population accounts for most of the ensemble reactivations during these time periods. This trend generally holds true for all reactivations encountered during a recording session, as evaluated by the statistics for the magnitude of all of the corresponding trajectories during the actual events and their reactivations (see Fig. 9G–I for results on all responsive, intensity-sensitive and intensity-invariant populations). Interestingly, there is no temporal evolution among the invariant units driving the reactivation of these memory patterns (e.g. the sequenced reactivations of the general units, followed by the subgeneral units, does not occur).

### Validation of reactivation patterns

**Figure 10:**
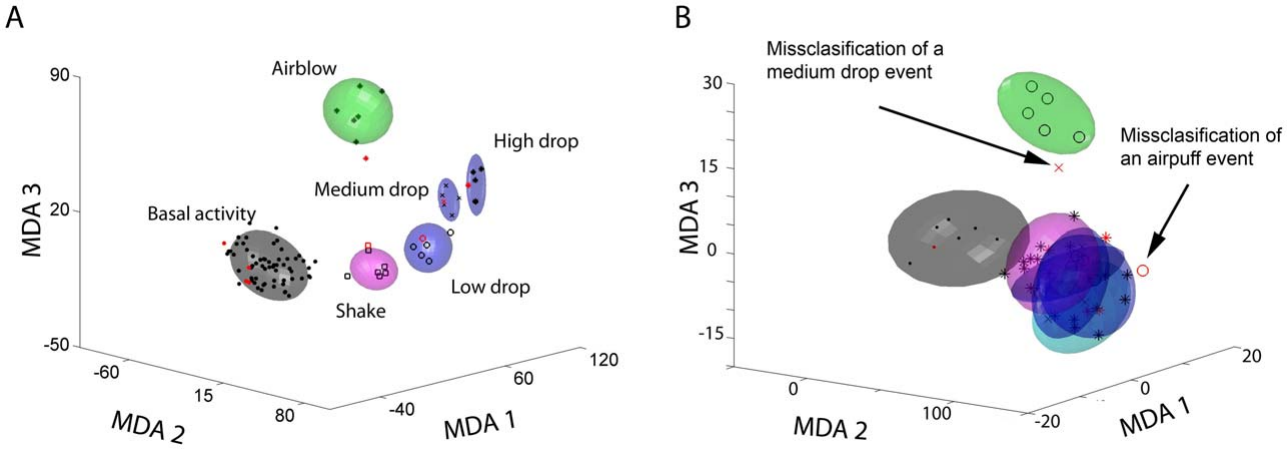
Shuffling of the data leads to the collapse of classification of population patterns. Scrambling technique revealed large overlap between the drop, shake and rest clusters (B) in comparison to the distinct clusters prior to shuffling (A). When the neural activities collected during events were shuffled among different units, the overall cluster structure collapses. In the case of air cluster, data shuffling leads to the misclassification and poor prediction of the test data (see the open circle symbol from an air-blow testing data was totally misclassified).

To ensure that our MDA analysis truly captured the CA1 encoding traces as well as reactivation traces, we carried out a set of control and validation tests. First, we determined the class membership for these test data points, which was based on the proximity to the clusters corresponding to the training data points. For example as shown in Figure 10A, a collection of 10 test data points (5 random startle points for air-blow, low drop, medium drop, high drop, and shake, as well as their corresponding 5 rest samples) was used to cross-validate the predictive power of models constructed using all other points (training). Average performances for class prediction were obtained by repeating MDA 1000 times with random partitioning into training and test points (Figure 10A). We noted that in general, the prediction performance is strongly correlated with the number of startle-responsive cells in the datasets, a feature that relates to the robustness within the encoding population.

As expected, when the neural activities collected during events were shuffled among different units, our cluster structure collapsed (see Figure 10B). As a result of this shuffling, the drop and shake clusters have a very large degree of overlap, in comparison to the original classifications prior to data shuffling, thus greatly diminishing the classification power. Even in rare occasions in which a distinct representation (e.g. one air-blow, and one drop event) could still be computed from a subset of the shuffled training data, it was apparent that the classification was incorrect. For example, one air blow or one medium drop event were totally misclassified (see these two misclassified points with arrows in the panel of Figure 6B). The data scrambling technique, therefore, demonstrates that the MDA discriminating power would be lost if the data were shuffled and any random change in the population activity could not fall into 5 distinct categories.

**Figure 11:**
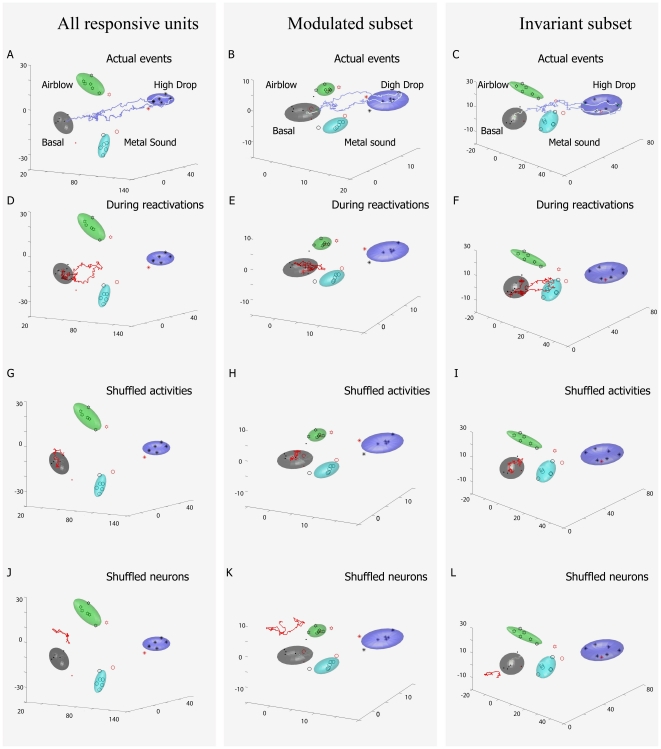
Shuffling techniques illustrate the specificity of the encoding patterns during learning and reactivations. We shuffled the spike responses of the top 50 invariant units during a drop reactivation using a time window of 2 seconds before and after the occurrence of the putative reactivation (by placing the spikes in 50 ms time bins and performing random permutation of the whole sequence). As a result, the projected trajectories are now located mainly inside the rest clusters (6G, H and I, are listed). The prior to shuffling was presented in panel A-F for comparison.

Because of the statistical classification that was used, the directionality and shape of the trajectories in MDA subspaces now provide a highly valuable means to compare and visualize the encoding patterns with any other random events [10]–[12], [22]. We further examined the effect of random changes of activity by shuffling the data specifically duration reactivations and then plotting the transient trajectories using sliding-window technique. In the first case (see pre-shuffle data in Figure 11A–F and the shuffled data in Figure 11G–I), we shuffled the spike responses of the top 50 invariant units during a drop reactivation. We focused on a time window of 2 seconds before and after the occurrence of the reactivation and we shuffled the spike times uniformly during the 4 second time interval. This was implemented by placing the spikes in 10 ms time bins and performing random permutation of the whole sequence within a given cell. As a result, the projected reactivation trajectories produced by shuffled data were found to be located mainly inside the rest clusters (Figure 11G for whole population, 11H for the intensity-modulated neurons, and Figure 11I for Invariant neurons).

The second method we used for shuffling reactivation data was to switch the identity of the neural units randomly during this putative reactivation. As illustrated in Figure 11J–L, this led to large trajectories that wandered out into unusual portions of the space regions unassociated with any pre-defined event categories. These two data-shuffling analyses have demonstrated the validity of MDA-sliding window method for elucidating ensemble encoding traces as well as reactivation traces. In other words, the statistical classification achieved by MDA methods, as described by the directionality and shapes of the transient trajectories, can provide a highly valuable means to compare and visualize the ensemble traces at both learning and post-learning periods.

**Figure 12:**
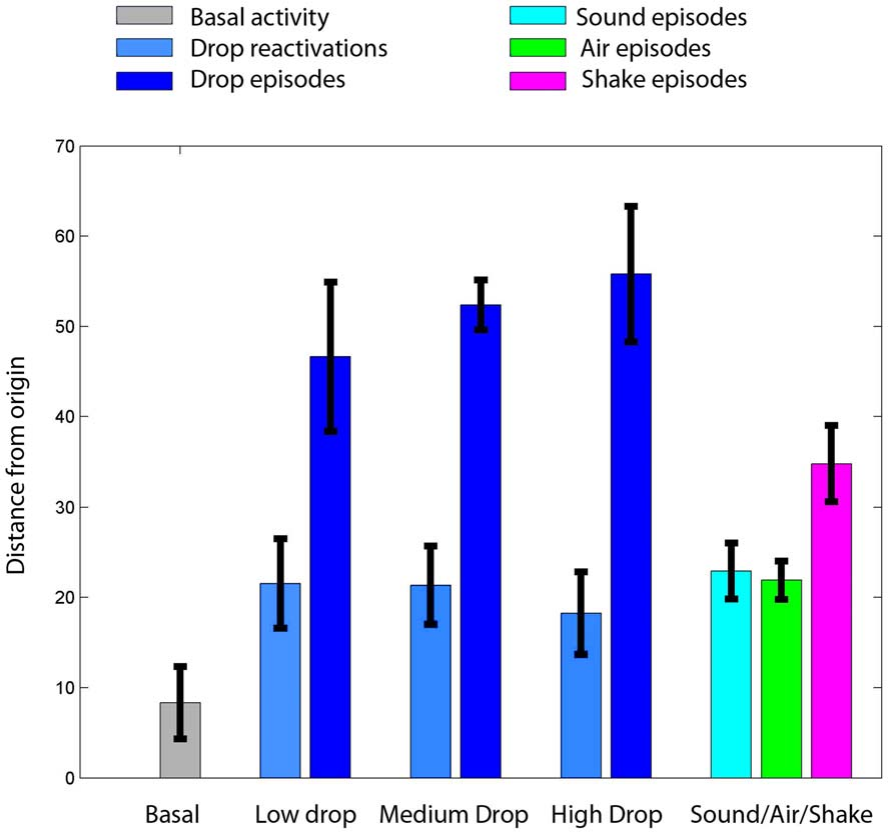
Similar magnitudes of reactivated trajectory distances among drop ensemble traces following low, medium, and high drop. The MDA distances were used as a way for averaging the mean of reactivated trajectories. The distances from sound, air puff, and shake clusters to the rest cluster center were also listed on the right side of the plot.

In addition, we further analyzed the relationship between event intensity and the amplitudes of reactivation traces. Interestingly, we have noticed that the magnitude of ensemble reactivations tends to remain more or less constant regardless the original event intensity. For example, we saw that high drop and low drop events produced similar magnitudes of reactivation trajectories of the episodic stimuli (Figure 12).

### Intensity-invariant neurons exhibit elevated correlation during reactivations

**Figure 13:**
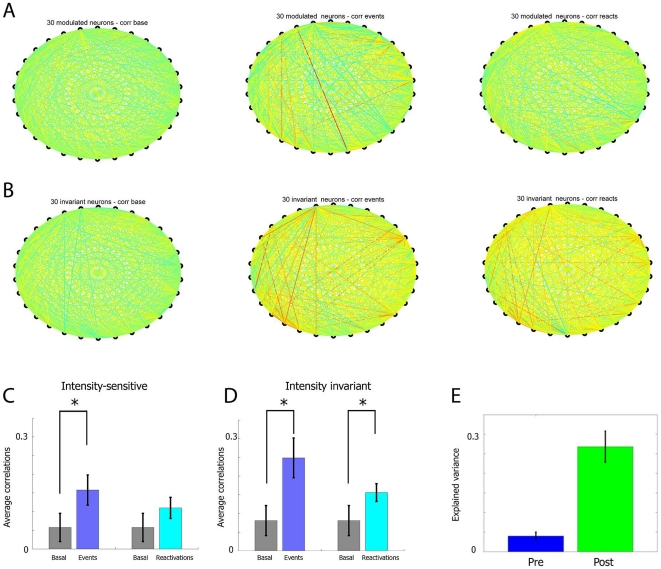
Correlations among intensity-sensitive and intensity-invariant neurons during basal, event and reactivation time periods. (**A**) Basal states correlation coefficient among 30 top neurons belonging to specific, subgeneral and general intensity-sensitive units (each category contains 10 cells) are displayed as colored lines that unite these 30 unit (left panel). Bluer colors indicate negative correlations and redder colors indicate positive correlations. While a significant number of units are more correlated during startle events (middle panel), these correlations mainly return back to basal values during reactivation time periods (right panel). (**B**) In contrast, for intensity-invariant neurons (10 for each of the specific, subgeneral and general categories, for a total of 30), although basal states are also characterized by low value for the magnitude of correlation coefficients (left panel), and by high values during the startle events (middle panel), these units remain highly correlated during the reactivation time period (right panel). (**C**) Statistics of the 254 pairs for intensity-sensitive group, which have been obtained by eliminating the pairs that are very weekly correlated during non-basal time periods (magnitude less than 0.05) are in agreement with the visual information displayed in A, indicating that the values during event time period are significantly different from the base levels, but the correlation levels during reactivation are not. (**D**) In contrast statistical analysis of the 315 pairs for the intensity-invariant group (obtained by excluding the correlations that have a magnitude less than 0.1) indicate that correlations are elevated during both events and time reactivation. (**E**) Explained variance during the time period before the stimuli and the time period after the stimuli have been delivered also suggest that correlations among units remain at higher levels in the post-stimuli time period, which is in agreement with the heightened correlation among intensity-invariant neurons.

To further confirm and explore the nature of the pattern reactivation, we applied two widely used methods, namely, pair-wise correlations [16], and explained variance [24]. Using the pair-wise cross-correlation method, we systematically assessed the coordination levels in firing changes among neurons belonging to either the intensity-modulated population or the intensity invariant population. We first analyzed the joint-firing tendency among the intensity-modulated neurons before, during, and after the episodic events (see Figure 13A for correlation graphs examples from mouse #1). Due to the intrinsic limitation of pair-wise correlation in terms of presenting the large neuron pairs, we plotted here a pooled set of 30 neurons containing the top best neurons from each class to visually illustrate how these top neurons' cross-correlation change over experiences. As expected, a significant number of neurons showed significant increase in their correlation during stimulus presentation (Figure 13A and B). However, the heightened cross-correlations among intensity-modulated cells largely came back to the basal levels once the episodic stimuli ended (Figure 13A). This is consistent with our MDA analysis that the intensity-modulated cell population contributes to the process of encoding, but is inactive during re-emergence of reactivation patterns. In contrast, the same kind of cross-correlation analysis revealed that in general the invariant cells exhibited significantly more correlated activity during the immediate post-event time periods (see Figure 13B for correlation graphs for the intensity-invariant neuron groups during basal, event and reactivation time periods, respectively). This, again, is in line with the MDA observation that the intensity-invariant units remained significantly more active during the putative reactivations than did the intensity-modulated groups. For the larger population of neuron pairs whose the minimal activation correlations were above a set threshold value of 0.05 (315 pairs for the intensity-invariant group and 254 pairs for intensity-modulated group), similar results were observed. That is, overall the intensity-modulated cells did not exhibit statistically significant increase in the cross-correlation (Figure 13C), whereas the intensity-invariant cells had the elevated increase in their co-firing tendency during the post-event reactivation period (Figure 13D).

Second, we also applied the Explained Variance analysis [24] and computed the correlation coefficients for each of the three conditions: pre, run and post session and followed by a regression analysis. This method essentially computes the correlation between correlation coefficients from correlation pairs in pre-event and event, pre-event and post-event, also event and post-event. We calculated Expected Variance (EV, see methods) for the data set from the reactivation period shown in Figure 13B (right side sub-plot). This analysis shows that the post-event value of EV is 0.268±0.04 in comparison to the pre-learning period (basal firing) value of 0.04±0.01 (Figure 13E). The explained variance analysis further confirms the elevated cross-correlation during the reactivation period for these invariant neurons.

**Figure 14:**
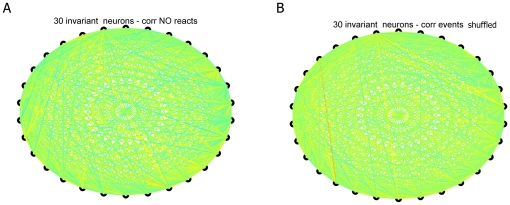
Comparison of correlations during reactivations, outside reactivation and during reactivation with shuffled neural activities. (**A**) No weak correlation coefficient among 30 top neurons belonging to intensity-invariant units outside reactivation periods; (**B**) no significant correlation with shuffled neural activities.

While these two cross-correlation based methods confirmed our MDA observation on the preferential reactivations by intensity-invariant cell population, we performed two additional tests: 1) We compared the cross-correlations between the time points at which MDA analysis detected putative reactivations (during a 2 sec before and 2 sec after the reactivation event) vs. the post-event time points at which trajectories were absent; and, 2) We shuffled the spike data specifically associated with transient reactivations of MDA trajectories. In the first test, it is expected that pair-wise correlations between cells should be more similar between the events and the subsequent “reactivations” time points. In contrast, these correlations should become much less similar during randomly chosen time periods of similar length chosen from the non-reactivation time points during the same post-event period. Indeed, our analysis showed that correlations within the invariant cells during the post-event non-reactivation periods showed little increase (Figure 14A). Furthermore, when we shuffled the invariant cells' spike data at those reactivation time points with other randomly chosen cells' spike data from the same period, the computed correlations among the shuffled spike trains were reduced to values close to zero for the whole population (Figure 14B). Thus, the above analyses strongly suggests that the elevated correlation is indeed derived from these intensity-invariant cells during the reactivation time points detected by MDA methods.

### Stronger correlation among invariant cells encoding general features

**Figure 15:**
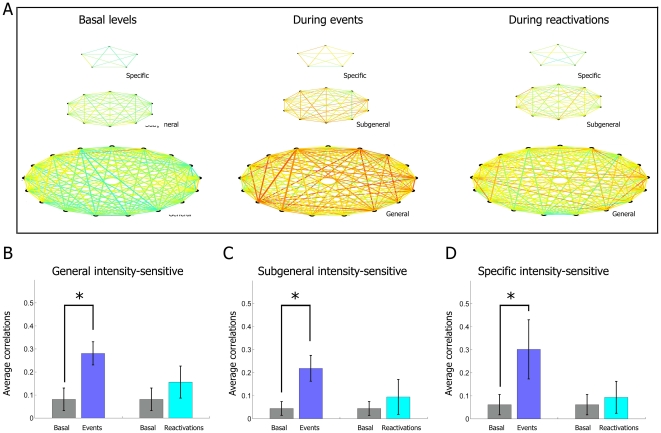
Cross-correlation analysis of the intensity-modulated subpopulation. (**A**) Correlations between top representative intensity-modulated units from mouse #1 encoding a specific type of event (top row), subgeneral features (multiple but not all types, second row), and general features (response to all types of events, third row), are displayed as colored lines during basal activity periods (first column), the actual event periods (second column) and at the time of reactivations (third column). High and low correlations are plotted with red and blue lines, respectively. (**B**) While the correlations increase significantly for the general-encoding and intensity-sensitive subpopulation during the startle episodes, these correlations return to values that are close to the baseline correlations. This trend is also manifest for the (**C**) subgeneral-encoding and intensity-sensitive and (**D**) specific encoding and intensity-sensitive subpopulations.

**Figure 16:**
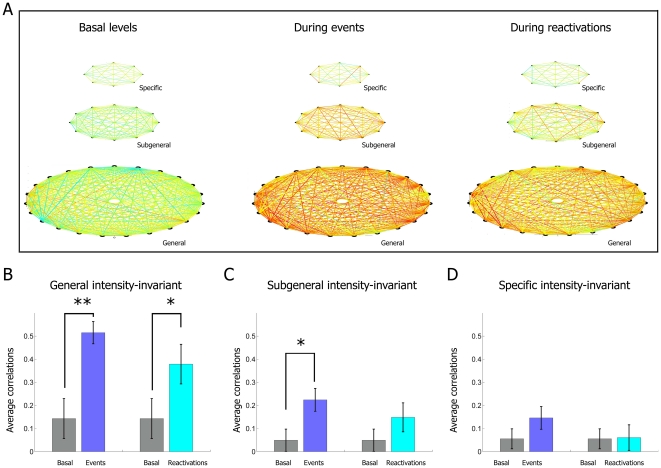
Cross-correlation analysis of various feature-encoding units belonging to the intensity-invariant subpopulation. (**A**) Correlations between top representative CA1 invariant units from data set #1 encoding a specific type of events (top row), subgeneral feature (two or more types of events, second row), and general feature (response to all four types of events, third row), are displayed as colored lines during basal activity periods (first column), the actual event periods (second column) and at the time of reactivations (third column). (**B**) In contrast to the intensity-sensitive results, significant average correlations are maintained during the reactivation period for the general intensity-invariant subgroups. These trends are maintained, although to a smaller degree, for the Subgeneral intensity-invariant population (**C**), and are not statistically different for the specific intensity-invariant subpopulations, although the very small sample size makes it impossible to draw any strong conclusion from this particular case (**D**).

Since we showed earlier that intensity-modulated cells can be divided into a set of subpopulation based on their response selectivity to multiple events or a specific event, we further investigated the levels of firing correlations among the intensity-modulated cells encoding general, subgeneral, and specific features during the reactivation periods (Figure 15A). As expected, while intensity-sensitive units were also characterized by heightened cross-correlation during the encoding period, these correlations decreased dramatically during the post-event reactivation period, regardless of the particular cell type (general, subgeneral or event-specific subgroups, Figure 15B–D). In contrast, when following the same classification scheme, the intensity-invariant cells encoding general features showed the strongest correlation (Figure 16A and B), followed by sub-general invariant cells, whereas specific invariant cells had weak or no significant elevation in their cross-correlation during the post-event reactivation period (Figure 16C–D).

**Figure 17:**
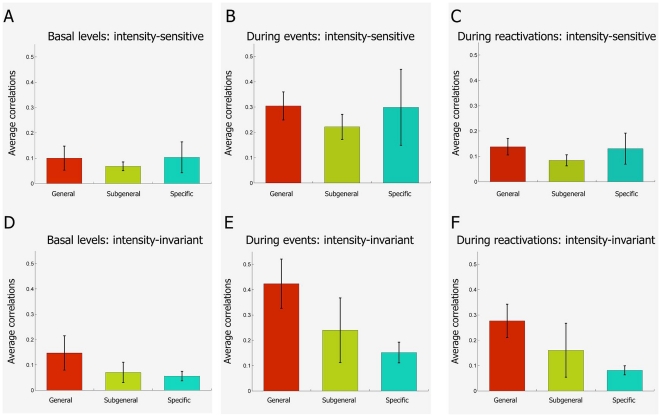
Cross-correlation analysis of various feature-encoding units belonging to the intensity-invariant subpopulation (the results were pooled from all 7 data sets). In agreement with results from data set #1, averaged correlations of various intensity-sensitive units pooled from all datasets from 7 mice increase from basal state (**A**), to higher values during the time of actual events (**B**), before they return to low/basal values during reactivation intervals (**C**) (p>0.05 for all conditions). In contrast, averaged correlations of various intensity-invariant encoding units not only increase from basal states (**D**) to higher values at time of actual events (**E**), but they also remain at elevated values during reactivations (**F**). These properties are stronger for the general units and weaker for the specific units, as compared to the subgeneral population (p<0.05 for all conditions).

The trends observed for the correlations among intensity-invariant neurons as well as among intensity-sensitive neurons are consistently manifest in the rest of our data sets. More specifically, using pooled data from 7 data sets, we showed that despite the increase in correlations for all groups of intensity-sensitive neurons from basal levels (Figure 17A) to event levels (Figure 17B), these correlations remain close to rest levels during reactivations (Figure 17C). In contrast, there are significant correlations among intensity-invariant neurons even during the reactivation time periods, although at weaker levels than during the actual startle episodes.

Further analyses of various subclasses of the invariant units suggest that the degree of correlation is strongest for the general-responsive units in comparison to the sub-general cells and the specific neurons (Figure 17D–F); Again, this is in line with the results from the individual data.

## Discussion

A hallmark feature of long-term memory consolidation is that only a portion of original information about various episodic events becomes long-term memory. Such information stored in the domain of long-term memory tends to be more general and abstract, and many specific details seem to be no longer available. Despite the many emerging studies which explore consolidation mechanisms at the molecular level [1]–[7], the neural mechanisms underlying this selective consolidation of episodic experiences have never been experimentally examined, thereby remaining completely unknown. Such selective consolidation and storage processes are widely assumed to be a part of normal forgetting process.

By taking advantage of recent large-scale recording and decoding methods [10]–[12], [21]–[25], here we designed a series of experiments to investigate how the hippocampal networks engages in such selective consolidation of long-term memory. Our present experiments provide several novel insights into how the hippocampal cell population may encode and consolidate episodic information. First, our parametric experiments demonstrate the existence of two distinct populations of CA1 episodic cells during the encoding of discrete episodic events: one for encoding sensory input intensity (Intensity-sensitive or Intensity-modulated cell population), and another for encoding mnemonic information independent of stimulus intensity (Intensity-invariant cell population). Second, the CA1 ensemble reactivation patterns were mostly derived from the intensity-invariant cell population. Third, within the intensity-invariant cell population there is an overall tendency that the invariant cells exhibiting general or broader responsiveness to multiple episodic events have much stronger firing cross-correlations than those of cells that respond only to a specific event. Currently, we do not know how the differential reactivations are influenced or modulated by behavioral and arousal states including stress, fear factors, and attentions [26]–[28]. These properties will need to be investigated in near future.

Our study on startling episodic events fits well with the reports that episodic hippocampal cells can undergo reactivations immediately after such events [10], [11] or trace fear conditioning [12]. The differential reactivations within episodic cell assemblies are quite interesting in considering cognitive significance. Moreover, such differential reactivation patterns have not been studied or described in place cell studies [16]–[20]. Place cells, by definition, encode on-going specific locations of the animals in a given environment, and therefore, may require different protocols to reveal such properties.

To provide moment-to-moment decoding of post-event real-time ensemble activity patterns, we have employed MDA/sliding window method to monitor and detect the temporal evolution of CA1 ensemble patterns during and after the startling episodic events. This dimensionality-reduction method has proven to be highly useful for intuitively visualizing the real-time transient dynamics and patterns associated with learning tasks [10]–[12], [21], [22], that is, our method has allowed us to pin-point the moment at which the ensemble patterns were reactivated. This MDA method provides intuitive visualization for its patterns (in terms of both the geometric shape and planar information of the transient trajectories) which can be further verified by two kinds of shuffled data analyses. It is important to point out that our analysis of firing sequences of individual episodic cells in the recorded data did not reveal any obvious sequential replay. This is not surprising since a given episodic event triggers co-activation of a unique cell assembly through which various groups of responsive cells simultaneously come together to encode different aspects of features of the event (from general to specific features) [10]–[12], [26]. In other words, a discrete startling event is represented by simultaneous co-activation of various episodic cells at that single moment.

Reactivations patterns of episodic experiences revealed by MDA/Sliding-window method were further corroborated by both pair-wise cross-correlations, and explained variance. In addition, we have found that preferential reactivations of distinct subgroups of intensity-invariant neuronal population seem to be responsible for ensemble pattern reactivations. More importantly, our analysis further revealed that during the reactivation time periods invariant, cells encoding general features of all or multiple events seem to be more correlated the cells encoding for specific events. This preferential reactivation mechanism may position the hippocampus toward extraction of generalized features from individual experiences of these events for long-term consolidation and storage. On the other hand, relatively weak cross-correlation among specific invariant cells may be a contributing factor in explaining why details of a given event tend to be more difficult to retain. Another contributing factor may be that such specific cells were detected in smaller percentages in our experimental data. This suggests that these cells may be prone to weakening of synapses over time during storage. This hypothesis needs to be tested in future experiments.

Our finding of preferential reactivations of cells encoding general and subgeneral features provides a strong experimental validation for the computational modeling work which simulates that the finer distinctions of specific events or knowledge may be more easily lost than the more general ones during the consolidation stage [29]–[31]. While the computation models were originally built to describe cortical binding function, our results suggest that such preferential consolidation properties are readily implemented at the level of the hippocampus.

Furthermore, our present study reveals a novel aspect of hippocampal consolidation principles unparalleled to those implied by the previous studies of place cell replays. Here, we show that hippocampal reactivations of episodic cell ensembles patterns are more than mere ‘imitation’ or ‘repetition’ of original patterns. In other words, the preferential and stronger reactivations of the invariant cells encoding general features enables the hippocampus to extract these major features of a given episodic experience and subsequently integrate them into the brain's general knowledge structure and semantic memory. This explanation may explain why patients with damaged hippocampi have great difficulty in forming episodic memories as well as concepts and general knowledge about these events.

Cognitively, episodic memory refers to memory of episodic events, and it is the major type of memory we encode in our daily life. In contrast, semantic memory refers to memory of facts and knowledge that are no longer ascribable to a particular occasion in life (without necessarily remembering where and when a person acquires it) [32]. Our recent discovery of the feature-encoding pyramid organizing principle in the hippocampus suggests an overall population-level mechanism for linking the formation of episodic memory with the emergence of semantic memory [11], [33]–[36]. Our present findings have further revealed how these two types of memories may be processed and organized through the regulation of neural network dynamics for long-term memory storage. It may also provide a new mechanistic framework for explaining and testing how semantic memory might be created through either single or repeated episodic experience [1], [3], [8].

In summary, our present study describes the subclassification properties among CA1 episodic cell assemblies encoding robust episodic events, and reveals their varying degrees of dynamic participation in post-event pattern reverberation and consolidation. Specifically, the event-intensity invariant CA1 neurons are largely responsible for the post-learning pattern reactivations. Moreover, during these transient reactivation periods, intensity-invariant cells encoding general features tend to exhibit stronger cross-correlations than do those cliques which encode specific features. Such post-event preferential reactivation of these general/subgeneral cell cliques may provide a key neuronal population-level mechanism for achieving the consolidation and storage of general information and knowledge in the brain.

## Materials and Methods

### Ethics Statement

All animal work described in the study were carried out in accordance with the guidelines established by the National Institutes of Health in the US regarding the care and use of animals for experimental procedures, and was approved by the MCG Institutional Animal Care and Use Committee at Georgia Health Sciences University (Approval AUP number: BR07-11-001).

### *In vivo* recording and spike sorting

We employed 96 and 128-channel recording arrays to record from the hippocampal region of freely behaving mice [10], [12], [21]. The multi-channel electrodes consist of two-independently movable bundles of stereotrodes or tetrodes, which were constructed by twisting a folded piece of 2 or 4 wires, respectively (STABLOHM 675, H-FORMVAR, 25 µm for stereotrode, California Fine Wire). After surgery, the mice were kept in their home cages for recovery for three to five days. The electrodes were then advanced slowly toward the hippocampal CA1 region, in daily increments of about 0.07 mm, until the tips of the electrodes had reached the CA1 region, as deduced from an assessment of field potential and neuronal activity patterns.

We subsequently recorded the ensemble activity from a large number of individual neurons during freely behaving states. The recorded spike activities from those neurons were processed in the manner as previously described [10], [12], [21]. Briefly, the spike waveforms and their associated time stamps for each of 128-channels were stored in data files using Plexon system format (*.plx). The artifact waveforms were removed and the spike waveform minima were aligned using the Offline Sorter 2.0 software (Dallas, TX), which resulted in more tightly clustered waveforms in principal component space. The Plexon system data files (*.plx) were then converted to Neuralynx system format (*.nst) and spike-sorted with the MClust3.3 program. This program permits classification of multidimensional continuous data. Its cluster splitting feature yields superior accuracy in comparison to the other available spike-sorting software and is therefore particularly suitable for spike sorting of hippocampal signals.

Principal component analysis was used to extract defining features from the spike wave shapes that were then used as part of the input for the MClust3.3 spike sorting program. The first two principal components, as well as the peak height, valley value, FFT and total energy of spike waveform parameters were calculated for each channel, and units were identified and isolated in high-dimensional space through the use of an autoclustering method (KlustaKwik 1.5) [37]. After autoclustering, the clusters containing non-spike waveforms were deleted using ‘KlustaKwik Selection’ function, and then the units were further isolated using a manual cluster cutting method in MClust (see an example in Figure 1). Only units with clear boundaries and less than 0.5% of spike intervals within a 1 ms refractory period were included in the present analysis. At the end of experiments, the mouse was anesthetized and a small amount of current was applied to four channels in the microdrive to mark the positioning of the electrode bundle. Histological Nissl staining (NeuroTrace® blue fluorescent Nissl stain) was used to confirm the electrode positions.

### Parametric changes in intensity of startling stimuli

We exposed mice to four types of robust episodic events: 1) a short and loud acoustic startle (intensity 85 Db, duration 200 ms), 2) A sudden air-blow to the animal's back (termed Air- Blow, 10 p.s.i); 3) A sudden drop of the animal inside a small elevator (termed Elevator-Drop, vertical freefall height); and 4) A sudden shake-like cage oscillation (termed Shake, 200 ms; 300 rpm). To maintain the consistency of stimulus inputs and yet minimize possible prediction of upcoming stimuli, the stimuli were triggered using a computer and delivered for seven times at randomized intervals within a few minutes. We previously showed that seven repetitions are sufficient for obtaining an adequate sampling of the neural responses, while minimizing the risk of habituation to the noxious stimuli [10], [11]. We varied the intensity of two types of episodic stimuli by changing the height of the drop (5, 13 and 30 cm) and the amounts of the air that were blown (200, 400 and 800 ms). The other two startling stimuli, the starling loud sound and shaking of the cage, were delivered at fixed intensity.

In our experiments we started recordings 30 minutes before a series of startling episodes were delivered to the mice. Each given type of startling event (e.g. Drop with a fixed height or Air puff with a fixed duration) was delivered in a single session for seven times with pauses ranging from 1-to-3 minutes at randomized intervals (inter-trial-intervals). A single event session lasted for about 20 minutes and the mice were then brought back to home cage for a brief rest for 5-to-10 minutes. This was followed by a different session consisting of either different startling events or the same event but at a different intensity (inter-session-intervals). All together, the mice would undergo three sessions of the same events (say Drop events with three chosen heights), plus two additional and distinct event sessions (e.g. Airpuff and Shake). Our typical experiments thus consisted of five event sessions which lasted for about 2.5 to 3 hours. The randomized inter-trial-intervals (1–3 minutes) were intended to minimize possible habituation and reduce the animal's ability to predict its upcoming event. We recorded population activity patterns in the CA1 region of the hippocampus from seven freely-behaving mice that were subjected to the following set of episodic stimuli: acoustic startling sound, cage shake, air-blow and elevator drop.

### Characterization of unit responses to startle stimuli

For our data analysis we selected only clearly separated single units that remained stable throughout the duration of the whole experiment. To select the responsive units, we first evaluated the changes in the firing frequencies in time bins of 500 ms immediately after the event start. The width of the time bins used here is appropriate to characterize the overall frequency changes after a startle event. To facilitate comparison between neurons that exhibit different increases/decreases over baseline activities, we used the transformation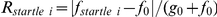. Here **f_startle i_** and **f_0_** represent average frequency responses during startles of type **i** or rest states, and **g_0_** is the average population activity during rest states. We maintained only the units which have high scores based on this metric. Note that this transformation allows for uniform quantification of the significant changes in firing patterns for units with both low- and high baseline firing rates. More precisely, changes in responses of low-firing units are proportional to absolute firing rate changes (since f_0_ ≪ g_0_), while response differences for the high-baseline units become proportional to the relative changes from the baseline activities (since f_0_ ≫ g_0_).

### Hierarchical clustering

Similar to our previous research [10], [22], we employed hierarchical clustering methods to investigate the structure of our neural data. We briefly outline the procedure here. We start by defining N clusters, one for each initial vector containing the responses to all types of startle stimuli. At each step, we proceed by uniting the two closest response vectors, or after a few steps, two closest groups. The two vectors or groups are merged into a new cluster and its mean is re-computed. These steps are then repeated and the nearest-neighboring groups are successively merged until they eventually form a single group. At each intermediate step of this procedure, the two clusters to be merged are aligned and linked at their best matching endpoints, forming a larger group.

### Projection analysis methods

We then used Multiple Discriminant Analysis (MDA) projection methods to classify the neural responses corresponding to different episodes into different classes [10], [12], [22]. Projection analysis methods are powerful tools that are well-adapted to deal with the complexity of large neural data sets data sets. These methods generate an encoding subspace of low dimension (on the order of number of classes). The use of these projection methods is particularly useful in revealing the inherent hierarchical structure that may exist in large-size neural populations.

To account for transient changes that may occur immediately after the startle events, we computed firing frequencies (f) in two 500 ms time bins immediately after the delivery of the stimuli. Baseline activities were characterized by computing the average firing rates during time intervals preceding the startle stimuli. We set aside randomly chosen population activities from one of each type of startle stimuli; this constitutes our test data set. The rest of the sampled population activities were then used to train our MDA statistical model. The matrix of mean responses during each category (rest and startle states) were then computed and used to compute the between-class scatter matrix 
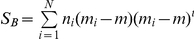
 [22]: Here **n_i_** is thenumber of elements in each class, **N** is the number of classes, **m_i_** is the mean vector for each class, **m** is the global mean vector and the symbol t indicate the transpose operator. To take into account the variations occurring for each class we also computed the within-class scatter matrix **S_W_**, which is defined as: 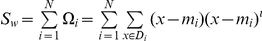. Here **D_i_** represents the set of population responses triggered by the **i** ^th^ startle type. Using these two matrices, it follows that a set of at most **N-1** discriminant projection vectors can be determined by computing the eigenvalue decomposition of the matrix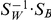.

For our data sets, the class covariance matrices **S_W_** were non-invertible, which is a direct consequence of data under-sampling, since the number of recorded neurons is much higher than the number of repeated trials. In practice, the matrix **S_W_** can be rendered invertible using a regularization technique which changes each class covariance matrices based on the following formula: **Ω_i_'  = (1 - λ) Ω_i_ + λ I**, where **Ω_i_** is the covariance matrix for the **i** ^th^ class, **λ** is a regularization parameter between 0 and 1, and **I** is the identity matrix. We determine the parameter **λ** automatically for each data set based on the optimization procedure we developed previously; each particular choice is determined by the particular distributions within each data set [22].

After computation of the **N – 1** discriminant dimensions is computed, we projected the neural patterns during startle episodes in this low-dimensional encoding subspaces. We then used the multivariate Gaussian distribution probability functions (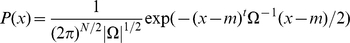) to fit the projections for each class. We subsequently enhanced our intuition about the relationships among classes by visualizing the 2σ boundary ellipsoids for each class. We tested the robustness of our MDA statistical model by employing different partitions of the training and test data points. In general we find that the performances for our model do not depend strongly on the particular choice of the training and test data selection.

In addition, we used a sliding window method to monitor the evolution of the population state throughout the duration of the experiment and to identify the occurrences of patterns similar to the ones experienced during the episodic events [10], [12]. A putative reactivation is deemed to have occurred whenever there are trajectories of significant amplitudes from the rest cluster towards the corresponding startle cluster. Inspection of the clusters generated by our use of Multiple Discriminant Analysis technique indicates that all different types of stimulations, including the parametric ones, can be successfully classified.

### Projection analysis methods with Shuffled Input

To rule out that the statistical properties of our projection analysis methods are creating classifications out of random data sets, we carried out these methods on noisy data obtained through two shuffling procedures.

We first created an input data set where the spike activities are shuffled among neural units at all times during the experiment. When these data are used as an input to previously computed cluster representations, the trajectories corresponding to different startle and reactivation events are mapped in the regions of the MDA encoding subspace where no other trajectories have been observed when the correct input has been presented. As such, this is an indication that the previous reference points, namely the cluster representation, are no longer useful in describing the trajectory dynamics.

We then proceeded to create a second data set where the input has been manipulated temporally. More precisely, the spike activities of all units have been shuffled within in a 4 second time period among bins of 50 ms width. As a result of the loss of simultaneous changes in activities across the neural population, the projected activities are now located mainly inside the basal activities rest cluster. Together, these two tests using shuffled data indicate that there is information loss regarding the startle events and their reactivation, and that the statistical methods no longer have a meaningful interpretation when these data are used.

### Correlation analysis

Since the correlation parameters cannot be computed between units recorded from different animals, we restricted this analysis to data recorded simultaneously from a single mouse. In order to allow for uniform quantification of changes in correlation between units during different temporal intervals across multiple data sets, only the top correlation pairs from the simultaneously recorded neurons of each mouse were used to compute the statistics. More precisely, we used the 10/20/30 pairs for the specific/subgeneral/general units respectively, reflecting the increasing number of units recorded in each one of these categories.

We used the following formula for computing correlations between pairs of neurons:


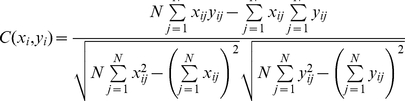
, where **N** is the number of repetitions for each startle stimuli, while **x_i_** and **y_i_** are the two vectors that contain the binned frequency responses during the **i** ^th^ repetition. The correlation coefficients were computed during baseline activities, or during the one second time intervals following the startle stimuli, using frequency sequences computed in 50 ms time bins (here x_ij_ indicates the binned frequency during the j^th^ time bin of the i^th^ startle repetition). To ensure that noisy correlation values are not included in the data sets, we set a low threshold (e. g. 0.05 or 0.1) and excluded them from the analysis.

To rule out the possibility that correlation results can be attributed to random changes in the neural population, we again used data sets where the spike activities were shuffled among neural units at all times during the experiment. Not surprisingly, all correlations among all neural units decrease to values close to zero in this case.

### Explained variance

We used the measure of Explained Variance (EV) [24] to further validate our cross-correlation analyses. We define the following three time periods: PRE, during the time interval prior to the startle events, EVENTS, during the startle events and POST, during the time period of putative startle memory reactivations. The EV value then is defined by:
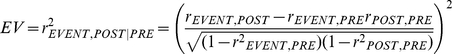
where the correlation coefficients can be obtained using the formula for computing correlations between pairs of neurons. A low value of EV would indicate that there is no learning effect attributable to the EVENT session (values are restricted between 0 and 1), while larger and larger values indicated stronger and stronger learning effects.
